# Supplementary material for: Mechanical Strength of Additive Manufactured and Standard Polymeric Components Joined Through Structural Adhesives
Source: Polymers (Basel). 2024 Oct 29;16(21):3036. doi: 10.3390/polym16213036 (PMC11548706; doi:10.3390/polym16213036)
Supplement: Supplementary file 1 [file polymers-16-03036-s001.zip › polymers-3269113-supplementary.pdf]

SUPPLEMENTARY MATERIAL

PVC – Uniaxial tensile tests

The tests on the PVC bars were carried out with the following characteristics:

- Galdabini SUN 500 uniaxial tensile machine
- Crosshead speed: 1mm/min of crosshead speed
- Specimen: 10mm diameter round PVC bar

Three replicates were considered, and the engineering stress strain curves are reported in Figure S1-a, while Figure S1-b reports an example of the specimen after failure. The yield stress and peak stress values are consistent, while the difference between the maximum strain is due to the material statistical variability.

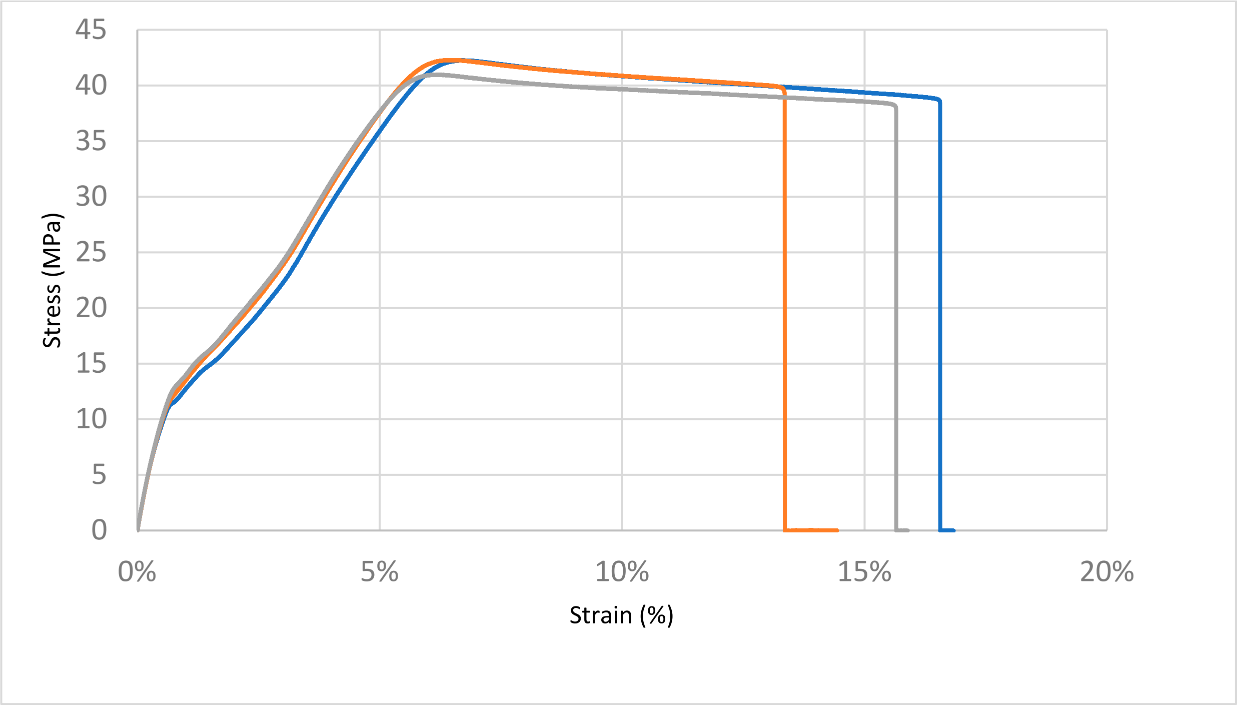

(a)

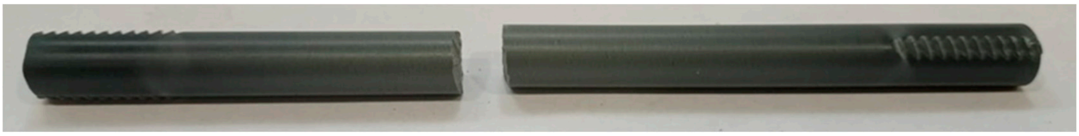

(b)

Figure S1- Uniaxial stress strain curves of three PVC bars (a) and typical specimen after failure (b)

ABS-AM – Uniaxial tensile tests on dog bone specimens

The tests on the additive manufactured ABS parts were carried out with the following characteristics:

- Galdabini SUN 500 uniaxial tensile machine
- Crosshead speed: 1mm/min of crosshead speed
- Specimen: dogbone specimens, based on ASTM D638 standard for plastic printed with a Stratasys Fortus 250mc with full dense option at maximum resolution

Three replicates were considered, and the engineering stress strain curves are reported in Figure S2. The yield stress and peak stress values are consistent, while the difference between the maximum strain at failure is due to the material statistical variability, which is higher for AM products due to their high presence of defects and surface imperfection.

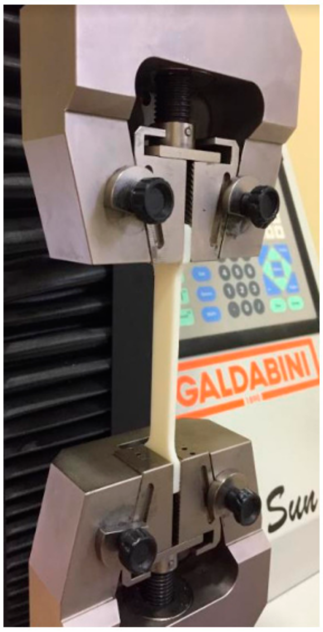

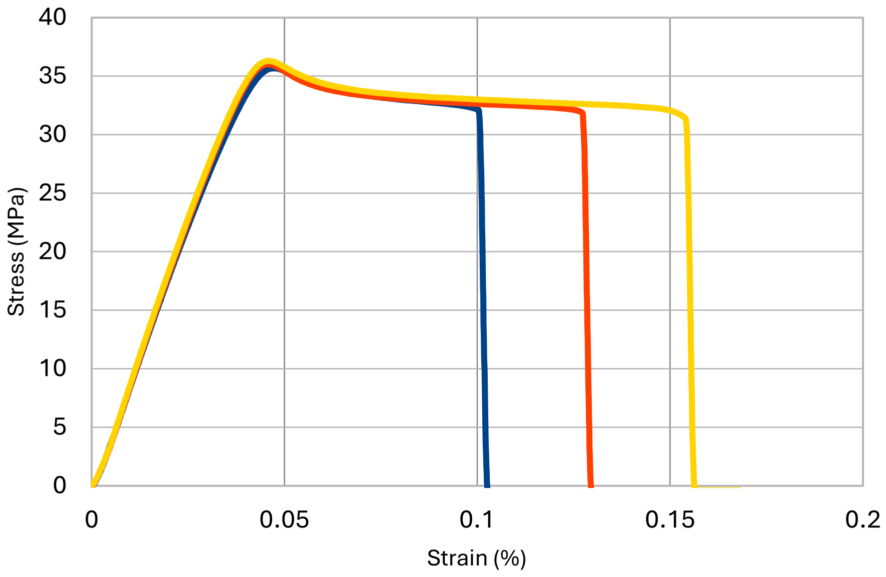

(a) *(b)*

Figure S2 – Uniaxial tensile test on three dogbone AM ABS specimens (a) and engineering stress-strain curves of the three samples tested
